# Validation of Two Rapid Diagnostic Tests for Visceral Leishmaniasis in Kenya

**DOI:** 10.1371/journal.pntd.0002441

**Published:** 2013-09-26

**Authors:** Jane Mbui, Monique Wasunna, Manica Balasegaram, Adrian Laussermayer, Rashid Juma, Simon Njoroge Njenga, George Kirigi, Mark Riongoita, Roberto de la Tour, Joke van Peteghem, Raymond Omollo, François Chappuis

**Affiliations:** 1 Centre for Clinical Research, Kenya Medical Research Institute, Nairobi, Kenya; 2 Drugs for Neglected Diseases initiative, Nairobi, Kenya; 3 Drugs for Neglected Diseases Initiative, Geneva, Switzerland; 4 Médecins Sans Frontières, Operational Centre, Geneva, Switzerland; 5 Geneva University Hospitals, Geneva, Switzerland; Institute of Tropical Medicine, Belgium

## Abstract

**Background:**

Visceral leishmaniasis (VL) is a systemic parasitic disease that is fatal unless treated. In Kenya, national VL guidelines rely on microscopic examination of spleen aspirate to confirm diagnosis. As this procedure is invasive, it cannot be safely implemented in peripheral health structures, where non-invasive, accurate, easy to use diagnostic tests are needed.

**Methodology:**

We evaluated the sensitivity, specificity and predictive values of two rapid diagnostic tests (RDT), DiaMed IT LEISH and Signal-KA, among consecutive patients with clinical suspicion of VL in two treatment centres located in Baringo and North Pokot District, Rift Valley province, Kenya. Microscopic examination of spleen aspirate was the reference diagnostic standard. Patients were prospectively recruited between May 2010 and July 2011.

**Principal Findings:**

Of 251 eligible patients, 219 patients were analyzed, including 131 VL and 88 non-VL patients. The median age of VL patients was 16 years with predominance of males (66%). None of the tested VL patients were co-infected with HIV. Sensitivity and specificity of the DiaMed IT LEISH were 89.3% (95%CI: 82.7–94%) and 89.8% (95%CI: 81.5–95.2%), respectively. The Signal KA showed trends towards lower sensitivity (77.1%; 95%CI: 68.9–84%) and higher specificity (95.5%; 95%CI: 88.7–98.7%). Combining the tests did not improve the overall diagnostic performance, as all patients with a positive Signal KA were also positive with the DiaMed IT LEISH.

**Conclusion/Significance:**

The DiaMed IT LEISH can be used to diagnose VL in Kenyan peripheral health facilities where microscopic examination of spleen aspirate or sophisticated serological techniques are not feasible. There is a crucial need for an improved RDT for VL diagnosis in East Africa.

## Author Summary

Visceral Leishmaniasis (VL) is potentially fatal if not treated promptly. Its diagnosis is based on the presence of parasites in spleen or bone marrow aspirates. These are invasive and risky procedures. Simple, rapid and non-invasive diagnostic tests are needed, notably in rural settings. We evaluated 2 rapid diagnostic tests, DiaMed IT LEISH and Signal KA for VL diagnosis, using splenic aspiration as the gold standard. The study was carried out in 2 hospitals located in Rift Valley province in Kenya, where VL is endemic. A total of 219 patients underwent splenic aspiration; 131 were positive and 88 were negative. DiaMed IT LEISH was able to correctly identify 117 of the positive cases, yielding a sensitivity of 89.3%, while Signal KA correctly identified 101, corresponding to a sensitivity of 77.1%. DiaMed IT LEISH was able to correctly label 79 of the 88 negative patients, yielding a specificity of 89.2%, while the Signal KA correctly labelled 84 of them, giving it a specificity of 95.5%. In conclusion, our study showed that rapid diagnostic tests can be used to diagnose VL in Kenyan rural health facilities, where splenic aspiration cannot be carried out safely.

## Introduction

Visceral Leishmaniasis (VL), also known as kala-azar, is a systemic parasitic disease transmitted through the bite of an infected phlebotomine sandfly. It is usually fatal if left untreated. This neglected disease affects mainly the poorest communities in Brazil, South Asia (India, Bangladesh and Nepal) and East Africa (Sudan, South Sudan, Ethiopia, Kenya, Uganda and Somalia) [1]. It is characterized by prolonged fever, anorexia, weight loss, splenomegaly, hepatomegaly, lymphadenopathy, as well as symptoms and signs of anemia, bleeding and concomitant infections (e.g. pneumonia). Given the lack of specificity of this clinical presentation and the significant toxicity and/or cost of current VL treatments, diagnostic confirmation is mandatory [2].

According to Kenyan national guidelines for VL, demonstration of parasite amastigotes by microscopic examination of smears from splenic aspirates is needed to confirm diagnosis and initiate treatment. Splenic aspiration is an invasive procedure that is highly sensitive (93.1–98.7%) but carries a small but significant risk of major bleeding [3], [4]. It should be performed by experienced clinicians in reference hospitals or research centers, and is therefore not suitable for use in first-line health services or district hospitals. Blood transfusion must be locally available in case of need. In addition, splenic aspiration is not possible in non-cooperative children, difficult in those with mild splenomegaly, and contra-indicated in persons with active bleeding, thrombocytopenia, severe anemia, as well as in pregnant and moribund patients. Identification of amastigotes in stained smears of splenic aspirates requires expertise and well-trained microscopists. Microscopic examination of lymph nodes or bone marrow aspirates is safer, but much less sensitive [4].

The limitations of parasitological diagnosis triggered the development of the Direct Agglutination Test (DAT) and, later, immunochromatographic rapid diagnostic tests (RDT) based of the detection of antibodies against the rK39 antigen [5], [6]. A meta-analysis on performance of rK39 RDT showed sensitivity and specificity estimates of 94.8% (95%CI: 92.7%–96.4%) and 90.6% (95%CI: 66.8% to 97.9%), respectively [7]. However, in this meta-analysis, rK39 RDT validation studies conducted in *Leishmania donovani* endemic areas were from South Asia and Sudan, and data were lacking from other endemic areas like Kenya and Uganda. Two rK39 RDTs were later validated in Amudat, Eastern Uganda, among a population of Kenyan and Ugandan Pokots. Whereas both tests showed high specificity (97–99%), the RDT from DiaMed AG, Switzerland, was more sensitive (97%) than the Kalazar Detect from Inbios, USA (82%) [8]. Moderate sensitivity of the Kalazar Detect in Ethiopia (75%), Sudan (78%) and Kenya (85%) was later confirmed in a multicentric study coordinated by the World Health Organisation (WHO) [9].

The Signal KA test is a recently developed and commercialized RDT based on the detection of antibodies against rKE16, a patented recombinant antigen from an Indian strain of *Leishmania donovani* [10]. This may in theory improve the diagnostic performance of the test, as the rK39 antigen consists of 39 amino acid repeats of a kinesin-like gene found in *Leishmania infantum/chagasi*. The test is commercialized by Span Diagnostics Ltd (Surat, India). Results of a phase II evaluation study in Spain showed the Signal KA test to be 92% sensitive and 99% specific in immunocompetent VL patients (n = 91), a diagnostic performance comparable with the rK39 Kalazar Detect [11].

As RDT diagnostic performance shows regional and brand-related variations, a local validation of the rK39 DiaMed IT-Leish and the rKE16 Signal KA was implemented among VL clinical suspect patients in two districts of the Rift Valley province in Kenya. The study results were meant to be integrated in the revised Kenyan national guidelines of diagnosis and management of VL.

## Methods

### Study design and procedures

This prospective phase III diagnostic study was conducted at Kimalel Health Centre, Baringo district, and Kacheliba Kala-azar Treatment centre, Pokot North district, both located in the Rift Valley province of Kenya, between May 2010 and July 2011. Patients ≥5 years presenting at the outpatient department with a history of fever for ≥14 days were examined for the presence of an enlarged spleen by one of the study physicians. VL clinical suspect patients, defined by a history of fever ≥14 days with clinical splenomegaly and malaria ruled out by a negative RDT (Paracheck), were eligible to participate in the study.

Baseline clinical data (symptoms, vital signs, weight, height, spleen and liver size) were collected in all participants. Five millilitres of blood were drawn to perform hemoglobin, leucocyte and platelet counts, prothrombin time index (PTI) and the two index tests: DiaMed-IT LEISH (DiaMed AG, Switzerland) and Signal KA (Span Diagnostics, India). The blood was centrifuged and the serum was collected. The Diamed-IT LEISH and Signal KA were performed on patients' serum according to standard operating procedures (SOP) following manufacturers' instructions.

Splenic aspiration was done by a clinician with extensive training and experience in the procedure after a systematic check for the absence of contra-indication(s): pregnancy, barely palpable spleen, active bleeding, jaundice, severely altered general condition, Hb≤5 gm/dl, platelets ≤40×103/UL, PTI difference between test and control ≥5 seconds. Two slides were prepared from splenic aspirates and were stained with Giemsa solution. The slides were read by an experienced laboratory technician who was unaware of the results of the index tests.

HIV testing was offered in all patients, as recently advised for RDT evaluation of VL diagnostics [12], and for optimizing patient care. Specific informed consent was obtained from the patient (or his/her parents or guardians for minors) before performing the HIV test. All patients who agreed to take an HIV test received pre- and post-test counseling. Subjects with positive HIV test results were referred to national HIV programs at Kacheliba district hospital or at Karbanet district hospital located 20 km from Kimalel.

### Quality assurance

A two-day refresher training workshop was organized at the Kenya Medical Research Institute (KEMRI), Nairobi, during the study preparation phase. Standard Operating Procedures were prepared and laboratory technicians from both study centers were trained to perform the two index RDTs, as well as for spleen aspirate staining and reading. All spleen aspirate slides from one study site were sent to the other study site for blinded re-reading. Slides with discrepant results between the two study sites and 10% of randomly chosen slides were read by an experienced parasitologist who was independent of the study.

### Data management and statistics

The demographic, clinical and laboratory characteristics of the patients were recorded on a case report form (CRF). Results of the index RDTs and of the microscopic examination of spleen aspirate were recorded in separate log books and later transferred into the CRF. Data was entered at KEMRI from copies of the CRF sent from the two field sites. Data analyses were done using the STATA software version 11 standard edition. Continuous data were summarized using mean and standard deviation or median and inter-quartile range when appropriate, while binary data was summarized using proportions. Comparison between VL and non-VL patients were made using t-test and Kruskall-Wallis test for continuous variables, or chi-square or Fisher's exact test for categorical variables. Sensitivity, specificity and predictive values of index tests were calculated using the result of spleen aspirate as the reference test, *i.e.* the test defining a VL and a non-VL case. Concordance between tests was determined using the Kappa index. The parameter estimates for specificity, sensitivity and predictive values are presented alongside their binomial exact 95% confidence intervals.

### Ethical considerations

The study was conducted in accordance with the ethical principles of the last revised version of the Helsinki declaration. Detailed information was made available for potential study participants in their language. A consent form was completed only after the patient had understood the points enumerated in the information sheet. Eligible patients were included after signing the informed consent form (or parent/guardian's for minors). Patients diagnosed with VL were treated according to the Kenyan VL national guidelines. Standard clinical care and free provision of VL diagnostic tests and drugs to all patients (whether or not enrolled in the study) were guaranteed by the participating centers. Non-VL patients were investigated and treated for alternative conditions by the study physicians. The study protocol was approved by the MSF Ethical Review Board on December 11, 2009 and by the KEMRI Ethical committee on March 30, 2010.

## Results

Of 251 eligible patients, 249 aged between 5–70 years were included in the study between May 2010 and July 2011; 2 patients refused to sign the consent form; 27 patients were excluded from analysis because of contra-indication(s) to splenic aspiration. Splenic aspirate slides from 10 patients (4.5%) with discrepant results between the two study sites were read by a third examiner for final classification. Three patients were excluded from analysis because of uncertain final diagnostic classification. Of 219 patients finally included in the analysis, 131 had a positive splenic aspirate (WHO parasite grading: 1–2: n = 16; 3–4: n = 52; 5–6: n = 63), defining VL, and 88 had a negative splenic aspirate, defining non-VL. No adverse-event following splenic aspirate was reported.

The demographic, clinical and laboratory characteristics of VL and non-VL patients are shown in table 1. VL patients were significantly less likely to be males (66% versus 80%, p = 0.024) and were more anemic (median Hb count: 6.9 versus 8.6 g/dl; p<0.001) than non-VL patients. HIV testing was done in 89% of patients and was positive in only 1 patient.

**Table 1:** Comparison of demographic and laboratory characteristics between VL and non-VL patients in Kacheliba and Kimalel, Rift Valley province, Kenya.

		Non-VL N = 88	VL N = 131	p-value*
Age (years)	Median (IQR)	16.5 (11;25)	16 (10;25)	0.110
Weight (Kg)	Median (IQR)	44 (29;51)	41.2 (25.1;50.8)	0.090
Sex (male)	n (%)	70 (79.6)	87 (66.4)	0.024
Haemoglobin (g/dl)	Median (IQR)	8.6 (7.3;9.7)	6.9 (6.2;8.3)	<0.001
Platelets (×10^3^/µl)	Median (IQR)	97.5 (68.5;128.5)	97 (70;127)	0.928
Spleen size (cm)**	Median (IQR)	10 (8;13)	11 (9;13)	0.544
HIV tests	Negative, n (%)	75 (85.2)	119 (90.8)	0.263
	Positive, n (%)	1 (1.1)	0	
	Not done, n (%)	12 (13.6)	12 (9.2)	

IQR = inter-quartile range.

* p-value from t-test or Kruskal-Wallis test for continuous variables and Chi-square or Fisher Exact test for categorical variables.

** measured between tip of spleen and left costal margin (at anterior axillary line level).

Out of 131 VL patients, the DiaMed IT LEISH was positive in 117 (sensitivity = 89.3%; 95%CI: 82.7–94%), whereas the Signal KA was positive in 101 (sensitivity = 77.1%; 95%CI: 68.9–84%). Out of 88 non-VL patients, the DiaMed IT LEISH was negative in 79 (specificity = 89.8%; 95%CI: 81.5–95.2%), whereas the Signal KA was negative in 84 (specificity = 95.5%; 95%CI: 88.7–98.7%). Sensitivity and specificity estimates, as well as negative and positive predictive values (NPV/PPV) of the two RDTs are summarized in table 2. The diagnostic performance of the two RDTs was not improved when used in combination, as all patients with a positive Signal KA test also had a positive DiaMed IT LEISH (data not shown). The agreement between the two index tests was 90.4% (90.7%; Kappa value = 0.815).

**Table 2:** Sensitivity, specificity, negative and positive predictive values of the DiaMed IT-LEISH and the Signal KA for VL diagnosis in Kacheliba and Kimalel, Rift Valley province, Kenya.

	Index test result	VL case	Non-VL case	Sensitivity	Specificity	PPV	NPV
		(N _total_ = 131)	(N _total_ = 88)	% [95% CI]	% [95% CI]	% [95% CI]	% [95% CI]
**Individual tests**							
DiaMed IT Leish	Positive	117	9	89.3	89.8	92.9	84.9
				[82.7 to 94.0]	[81.5 to 95.2]	[86.9 to 96.7]	[76.0 to 91.5]
	Negative	14	79				
Signal KA	Positive	101	4	77.1	95.5	96.2	73.7
				[68.9 to 84.0]	[88.7 to 98.7]	[90.5 to 98.9]	[64.6 to 81.5]
	Negative	30	84				

Abbreviations: PPV = positive predictive value, NPV = negative predictive value, CI = Confidence Interval.

## Discussion

Two RDTs were evaluated and compared in a prospective phase III study among VL suspect patients in the Rift Valley province of Kenya, using microscopic examination of spleen aspirate as the reference standard. The prevalence of HIV infection in the studied cohort was low (0.4%), an important consideration as sensitivity of most serological tests (with the notable exception of the DAT), including rK39-based RDT, is decreased in co-infected patients [13], [14]. The DiaMed IT LEISH was more sensitive (89.3%; 95%CI: 82.7–94%) than the Signal KA (77.1%; 95%CI: 68.9–84%), but the difference did not reach statistical significance. Combining the tests was not useful, as all patients with positive Signal KA had a positive DiaMed IT LEISH. A recent phase II multicentric evaluation of several RDTs in East Africa led to similar findings; in this study sponsored by WHO/Special Programme for Research and Training in Tropical Diseases (TDR), sensitivity of the DiaMed IT LEISH (87.2%; 95%CI: 82.5–90.8%) was significantly higher than the Signal KA (73.2%; 95%CI: 67.4–78.3%) [15], [16].

The specificity estimates of the DiaMed IT LEISH (89.8%; 95%CI: 81.5–95.2%) and the Signal KA (95.5%; 95%CI: 88.7–98.7%) were statistically comparable. As the prevalence of VL among clinical suspects was high in both treatment centers, PPV were high (93–96%) and both tests can therefore be used for VL confirmation, provided that they are strictly applied on rigorously defined clinical suspect patients. The PPV would indeed drop in settings where the prevalence of VL is lower among the population tested. It should be reminded that none of the existing serological tests for VL can be used to diagnose relapse due to the long persistence of specific antibodies following treatment of primary VL.

Spleen aspirate is one of the recommended reference standards for VL diagnostic evaluation studies [12]. Its sensitivity is high (≥95%), but false negative results do occur, which may have induced a mild underestimation of the specificity of the two RDTs in our study. Specificity of microscopic examination of spleen aspirate should in theory reach 100%, but it is slightly less in practice (*e.g.* due to suboptimal staining). Therefore, a certain degree of under-estimation of RDT sensitivity estimates cannot be excluded. Considering the stringent quality controls implemented in this study, we believe that patient misclassification was minimized.

The RDTs were performed on serum in our study, whereas they are likely to be used on whole blood from finger prick in clinical practice, especially at primary health care level. A recent study showed excellent concordance of results between blood and serum samples of an rK39 RDT in India [17]. These data are reassuring but a similar comparison should be done with other brands of RDTs in various locations, including East Africa.

In practice, RDTs will also be used in patients who do not strictly meet the case-definition of VL clinical suspect or who meet one or more exclusion criteria used in this study. As the diagnostic performance of RDTs for VL is not known for these subgroups of patients (e.g. with <2 weeks fever, minimal splenomegaly, age <5 years), a certain degree of spectrum bias in our study cannot be excluded.

In addition to diagnostic performance, other test characteristics are important to consider, such as sustainability of production, robustness, shelf-life, cost and ease of use, which were not formerly assessed in our study. Ease of use (clarity of kit instructions, technical complexity, ease of interpretation of results, time to results, equipment required) of the DiaMed IT LEISH and the Signal KA were rated as similar in a recent study [16]. Nevertheless, the cold chain requirement of the Signal KA (storage at 2–8°C) is a major obstacle to field use in most East African VL endemic areas. Prolonged exposure to very high temperatures (≥45°C) may also have a negative impact on the accuracy of the DiaMed IT LEISH [16].

Considering (i) the limited sensitivity of the Signal KA, the DiaMed IT LEISH and other commercialized RDTs in East Africa and (ii) the lethal characteristic of the illness if left untreated, VL cannot be excluded in clinical suspect patients with a negative RDT result in this region. In these patients, a more sensitive test such as the DAT or microscopic examination of spleen aspirate should be applied to confirm or rule-out VL. Unfortunately, these two procedures cannot be applied in Kenyan peripheral health structures without specific support from academic institutions or NGOs. In these remote settings, the DiaMed IT LEISH is currently the best available RDT for VL because its sensitivity is higher than the other commercialized RDTs [8], [15], [16]. It should be noted that the IT LEISH is now commercialized by Bio-Rad (Marnes-la-Coquette, France) but the product has remained the same.

The results of this study resulted in the integration of the DiaMed IT LEISH in the revised VL Kenyan national guidelines as first-line diagnostic test in settings where splenic aspiration or DAT cannot be done [18]. We believe that this decision will facilitate patients' access to improved VL diagnosis in the country. Nevertheless, a more sensitive RDT for primary VL diagnosis in East Africa remains deeply needed.

## Supporting Information

Figure S1 STARD flowchart of the study.
(DOCX)

Table S1 STARD checklist of the study.
(DOC)
