# Supplementary material for: Validation of Two Rapid Diagnostic Tests for Visceral Leishmaniasis in Kenya
Source: PLoS Negl Trop Dis. 2013 Sep 26;7(9):e2441. doi: 10.1371/journal.pntd.0002441 (PMC3784478; doi:10.1371/journal.pntd.0002441)
Supplement: Figure S1 — STARD flowchart of the study. (DOCX) [file pntd.0002441.s001.docx]

Non-VL

n=79

VL

n=14

Non-VL

n=9

VL

n=117

Spleen aspirate

n=127

DiaMed IT LEISH and/or Signal-KA Positive

n=127

Inconclusive

n=2

Inconclusive

n=1

Spleen aspirate

n=95

DiaMed IT LEISH or Signal-KA

Inconclusive

n=0

DiaMed IT LEISH and Signal-KA

Negative

n=95

DiaMed IT LEISH and Signal-KA

n=222

Eligible patients

n=251

Excluded patients:

Consent form not signed: n=2

Spleen aspirate contra-indicated: n=27
